# Determining and predicting biochemical disease trajectory in intrahepatic cholestasis of pregnancy: A longitudinal cohort study

**DOI:** 10.1177/1753495X251394433

**Published:** 2025-12-09

**Authors:** Nicholas J Williamson, Jenna Sajous, Tim IM Korevaar, Paul T Seed, Jenny Chambers, Peter H Dixon, Lucy C Chappell, Catherine Williamson, Caroline Ovadia

**Affiliations:** 1 Department of Women and Children's Health, 4616 King's College London, London, UK; 2 Department of Internal Medicine and Academic Center for Thyroid Disease, 6993 Erasmus University Medical Center, Rotterdam, The Netherlands; 3 Women's Health Research Centre, 4615 Imperial College Healthcare, London, UK; 4 Department of Metabolism, Digestion and Reproduction, 4615 Imperial College London, London, UK; 5 Centre for Reproductive Health, Institute for Regeneration and Repair, 47967 University of Edinburgh, Edinburgh, UK

**Keywords:** Intrahepatic cholestasis of pregnancy, predictive biomarker, bile acid, alanine aminotransferase, bilirubin

## Abstract

**Background:**

The severity of intrahepatic cholestasis of pregnancy (ICP) reflects peak maternal bile acid (BA) concentration. However, the course of the disease is unclear.

**Methods:**

Longitudinal observational cohort study of individuals with ICP. Serial BA and alanine aminotransferase (ALT) trajectories were determined according to starting severity. Multiple logistic regression identified variables predictive of subsequent disease severity, validated using data from a randomised controlled trial.

**Results:**

Although highly variable, BA concentrations increased across gestation (*p* > 0.001). Normal ALT concentration at diagnosis predicted concurrent non-severe disease (negative predictive value 94.2% (91.6–96.1%)). Gestational age and BA concentration at diagnosis somewhat predicted later moderate or severe disease (BA ≥ 40 µmol/L: ROCAUC 0.64 (0.58–0.69); BA ≥ 100 µmol/L: ROCAUC 0.68 (0.62–0.73)), similarly in the validation cohort (ROCAUC 0.70 (0.65–0.76) and 0.69 (0.63–0.76), respectively).

**Conclusion:**

An earlier gestation and higher BA concentration at diagnosis increase the likelihood of more severe disease; however robust prediction is limited.

## Background

Intrahepatic cholestasis of pregnancy (ICP) typically presents with pruritus and elevated serum bile acid (BA) concentrations. When BA rise above 40 µmol/L (moderate disease), the risk of spontaneous preterm birth, admission to the neonatal unit and meconium-stained amniotic fluid increase,^1^ and when peak concentrations are above 100 µmol/L (severe disease) there is an increased risk of stillbirth, irrespective of ursodeoxycholic acid (UDCA) treatment^2^; at presentation with ICP, it is not clear who will develop moderate or severe disease. Given that management decisions are dictated by peak BA concentration, this has become a relevant clinical outcome.

Mild increases in serum BA occur in normal pregnancy.^3^ However, the placebo-controlled PITCHES trial of UDCA suggested a gradual decline in serum BA concentrations for women with ICP with advancing gestation,^4^ contrasting with previous understanding.^5^ UDCA is the most commonly used disease modifier in ICP; UDCA replaces other hydrophobic BA in the BA pool, and comprises approximately 60% of measured BA in treated patients.^6^ Although its impact on itch severity is minor,^7^ UDCA reduces the risk of spontaneous preterm birth.^8^

We aimed to characterise the course of BA in ICP, separated by UDCA treatment; and whether alanine aminotransferase (ALT) and bilirubin are useful markers of BA concentration. We then aimed to identify patient characteristics at ICP diagnosis that could predict subsequent moderate or severe disease severity (BA ≥40 µmol/L, or ≥100 µmol/L).

## Methods

### Participant recruitment

Participants were recruited to a longitudinal observational study of ICP from nine UK hospitals and via the patient charity, ICP Support, from 1997 to 2020. The study was performed according to the Declaration of Helsinki, and was approved by the Ethics Committee of Hammersmith Hospital National Health Service Trust, London, UK (97/5197, 17/WA/0161 and 08/H0707/21). Participants gave individual informed written consent; summary data are available on request of the corresponding author; individual participant data can be shared where consent was indicated.

ICP was diagnosed according to hospital criteria at recruitment; predominantly raised non-fasting BA and/or liver aminotransferases in combination with pruritus, in the absence of other dermatological or hepatic causes. Maternal demographics, pregnancy outcomes, and serial serum measurements of BA, ALT and bilirubin concentrations were collected. For this study, we selected pregnancies where the first BA measurement was at or before 34 weeks'.

**Figure 1.**
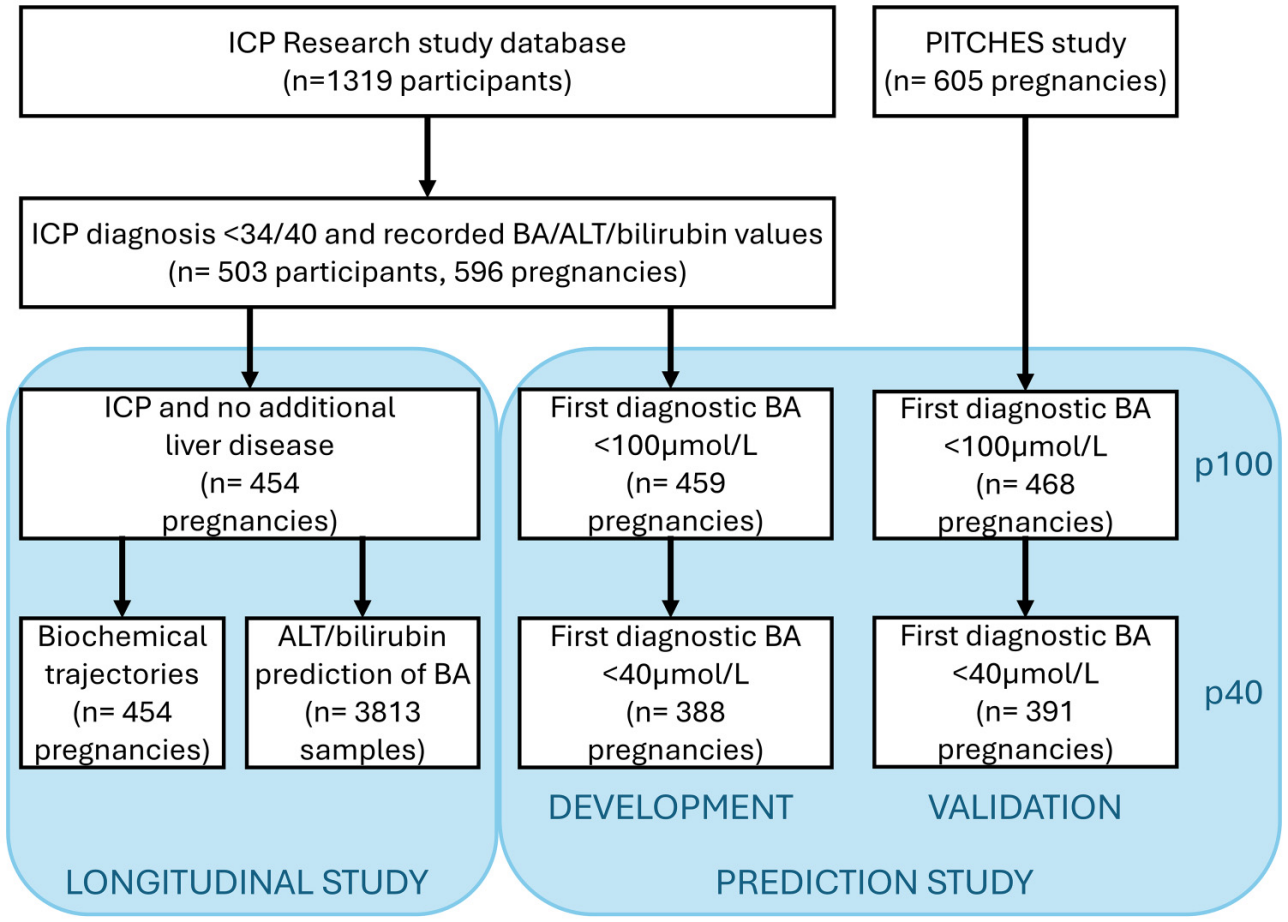
Participant inclusion in study components.

For the study of longitudinal BA trends, we restricted analyses to pregnancies with confirmed ICP excluding those with additional liver disease. For BA prediction, we included a more permissive dataset to reflect a pragmatic clinical approach where prediction of later BA elevation may be used for all patients with suspected ICP or other causes of gestational hypercholanaemia (Figure 1).^9^

Data from the PITCHES randomised controlled trial of UDCA versus placebo in ICP were used to validate the predictive model.^7^

### Longitudinal study

This study had two ‘end-points’ – characterisation of the longitudinal pattern of BA, ALT and bilirubin concentrations in ICP, and calculation of the sensitivity and specificity of raised ALT or bilirubin to predict moderate or severely elevated BA concentrations in concurrent blood samples.

BA concentrations were winsorized to account for the impact of outliers. We used linear mixed effects regression models to analyse the gestational trajectories of mean BA, ALT and bilirubin concentrations in the untreated and treated groups, using a random intercept per individual based on sensitivity analyses comparing models with various combinations of random intercepts and/or slopes using the Akaike information criterion and the Bayesian information criterion. A product interaction term of ‘gestational age*variable’ was introduced to test changes over time according to maximum total BA, ALT or bilirubin concentrations. Models were adjusted for maternal BMI, age, number of previous pregnancies and ethnicity. Analyses were performed using R statistical software version 3.03 (packages rms, MASS, hmisc and lme4, sjPlot and sjmisc; https://www.r-project.org/). The ‘diagt’ code in Stata was used to calculate the performance of ALT and bilirubin to identify concurrent elevations in BA concentrations (either moderate or severe disease).

### Prediction model

The ‘end-point’ of this study was to predict the ultimate severity of ICP (based upon peak BA concentration) at the time of first diagnosis. For analysis purposes, we treated each pregnancy as a separate event. To predict later BA concentration over 40 or 100 µmol/L, we removed patients who exceeded these thresholds at their first visit. Predictors were selected that were available at diagnosis, and were first considered individually. Fractional polynomial regression was used to check whether taking logs or other transformations improved the performance of the continuous predictors.^10^ Multiple logistic regression was used to combine the individually useful predictors into two predictive models – for maximum BA ≥40 µmol/L (pred40) and ≥100 µmol/L (pred100).

The pred40 and pred100 models were validated internally (using our training set) and externally (using the PITCHES trial participants). Observed event rates were compared to rates expected based on the average value of pred40 or preP100 using confidence intervals based on the binomial distribution (‘bitest’ code in Stata).

We used the Stata code ‘pmsampsize’ to estimate the appropriate number of variables to be used within development of the predictive model. With a fixed sample size based upon recruited participants, and C statistic 0.8, we determined that 11 and 13 inputs could be used in our pred40 and pred100 models, respectively.

## Results

### Trajectory of bile acid, alanine aminotransferase and bilirubin concentrations across gestation

**Figure 2.**
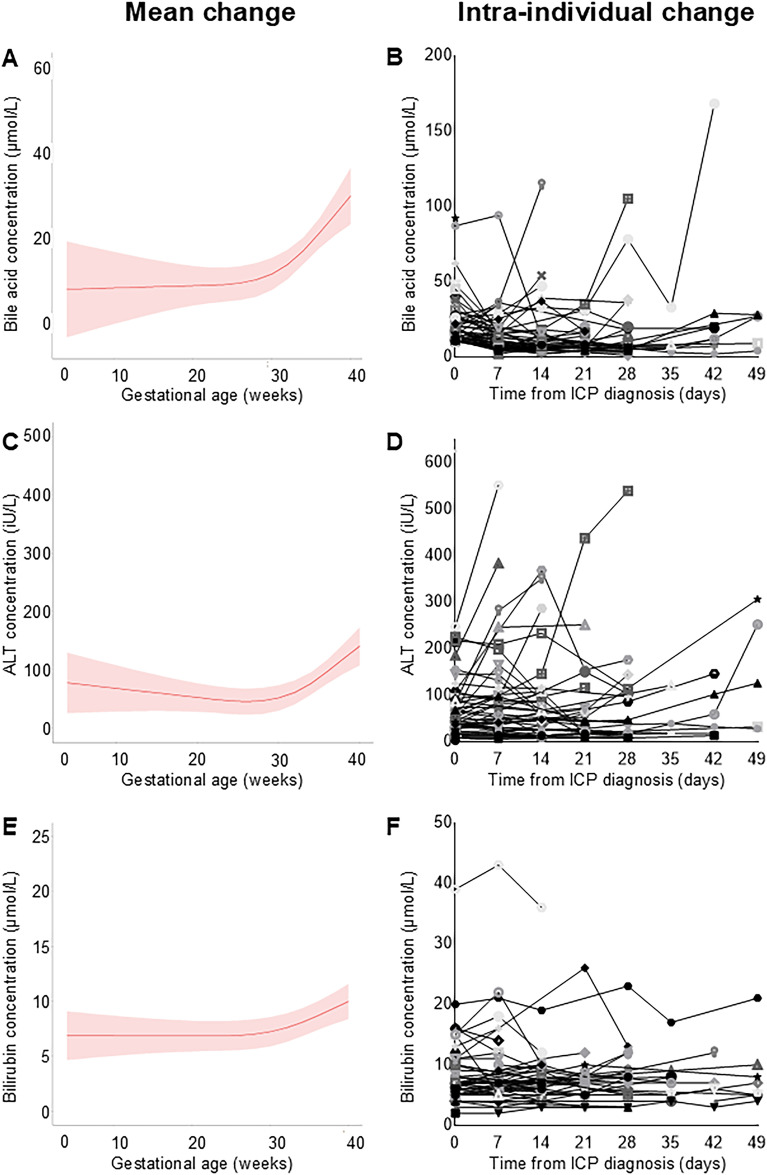
Longitudinal measurements of biochemical markers with advancing gestation in ICP, for patients not receiving UDCA or other disease modifying medications.

Data from 454 pregnancies were available for inclusion, of which 390 (86%) were treated with UDCA; patient details are summarised in Supplemental Table 1. Overall, mean serum BA, ALT and bilirubin concentrations increased with advancing gestation in patients not receiving UDCA (*p* < 0.001), however there was considerable interindividual variation (Figure 2). Irrespective of disease severity, BA concentrations fell after the first diagnostic measurement for 65.6% (42/64), this was sustained for 39.1% (25/64), and BA concentration normalised for 18.8% (12/64). Utilising more recently suggested higher diagnostic thresholds, 34.4% (22/64) would not have been diagnosed based upon BA concentration alone (non-fasting BA < 19 µmol/L).^11^

Bile acid (A, B), alanine aminotransferase (C, D) and bilirubin (E, F) serum concentrations, by gestational age (A, C, E) or days from ICP diagnosis (B, D, F). A, C, E show mean and 95% confidence interval; B, D, F show concentrations for individual participants, by value closest to each week, up to 49 days from diagnosis. Data from 64 patients are shown, 45 (70.3%) with mild, 15 (23.4%) moderate and 4 (6.3%) severe disease as per peak BA concentration. Of 59 with initial mild disease, 10 (16.9%) developed moderate, and 4 (6.8%) severe BA elevation.

**Figure 3.**
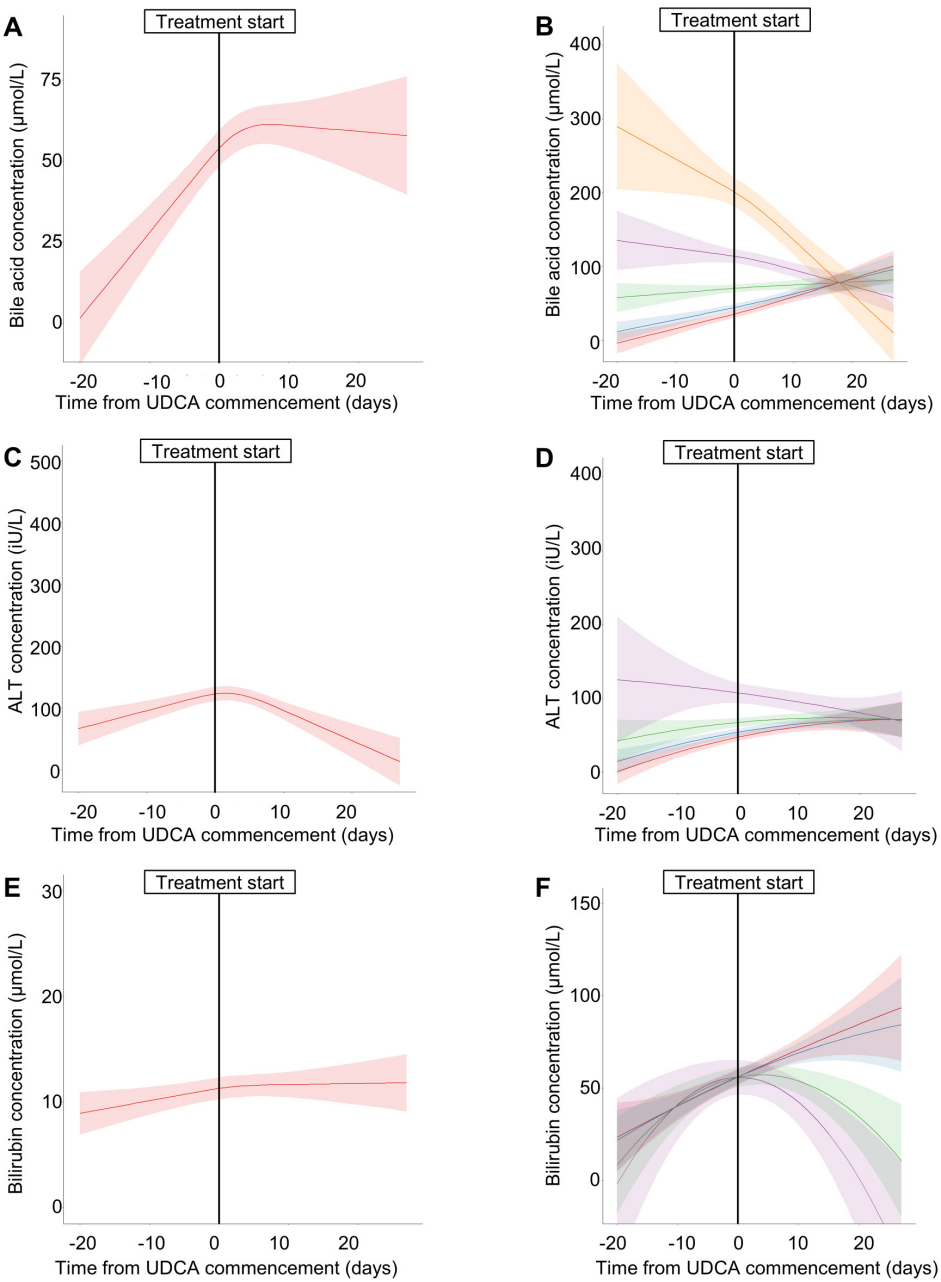
Longitudinal measurements of biochemical markers with advancing gestation in ICP, for participants treated with ursodeoxycholic acid (UDCA).

Patients treated with UDCA had more severe disease than those not receiving UDCA: 85 (21.2%), 163 (41.8%) and 142 (36.4%) had mild, moderate and severe disease, respectively. There appeared an overall trend for BA and ALT concentrations to reduce after commencing UDCA (Figure 3). However, when stratified by severity of BA/ALT elevation, individuals with the largest falls were those with the highest respective values at the start of treatment (implicating regression to the mean). In contrast, bilirubin concentration slightly increased following treatment when the values were less than 100 µmol/L (*p* < 0.03). Individual trajectories of BA concentrations varied widely between individuals (Supplemental Figure 1), and an initial increase following UDCA treatment occurred for 51.4% (165/321) within 14 days (Figure 3, Supplemental Figure 1).

Bile acid (A, B), alanine aminotransferase (C, D) and bilirubin (E, F) serum concentrations, by days from UDCA treatment. A, C, E show overall mean and 95% confidence interval for all concentrations; B, D, F show mean concentrations separated by concentration.

Concurrent measurements of BA, ALT and bilirubin were assessed: of 1324 samples from patients not receiving UDCA, 462 (34.9%) had normal ALT concentrations (<33 IU/L), of whom 4.1% (19) hadmoderately, and 1.7% (8) had severely elevated BA concentrations. Similarly, with normal bilirubin concentration (17 µmol/L), 97/1182 (8.2%) had moderate, and 29/1182 (2.5%) had severely elevated BA (Table 1). There were consistently higher rates of co-existent moderate or severe cholestasis for patients receiving UDCA with normal ALT or bilirubin concentrations. Although ALT and bilirubin did not closely predict concurrent moderate or severe BA elevation (ROC AUC 0.55 to 0.71), ALT elevations were more sensitive (75.8 to 85.7%), and bilirubin concentrations were more specific (93.1 to 96.2%). Particularly for patients not receiving UDCA, normal ALT had a high negative predictive power to detect BA concentration ≥40 µmol/L (94.2%) or ≥100 µmol/L (98.3%).

**Table 1.** Use of concurrent bile acid and alanine aminotransferase or bilirubin measurements to identify patients with moderate or severe hypercholanaemia in ICP.

	Treated with UDCA		Not treated with UDCA		All	
	ALT 33 IU/L	Bilirubin 17 µmol/L	ALT 33 IU/L	Bilirubin 17 µmol/L	ALT 33 IU/L	Bilirubin 17 µmol/L
Number of measurements	2489	2273	1324	1293	3813	3566
Number with normal LFT and moderate cholestasis	149/821 (18.1%)	486/1994 (24.4%)	19/462* (4.1%)	97/1182* (8.2%)	168/1283 (13.1%)	583/3176 (18.4%)
Number with raised LFT and moderate cholestasis	465/1668 (27.9%)	70/279 (25.1%)	109/862 (12.6%)	25/111 (22.5%)	490/2530 (19.4%)	95/390 (24.4%)
Number with normal LFT and severe cholestasis	82/821 (10.0%)	166/1994 (8.3%)	8/462* (1.7%)	29/1182* (2.5%)	90/1283 (7.0%)	195/3176 (6.1%)
Number with raised LFT and severe cholestasis	259/1668 (15.5%)	156/279 (55.9%)	48/862 (5.6%)	25/111 (22.5%)	307/2530 (12.1%)	181/390 (46.4%)
Sensitivity to diagnose moderate ICP	75.8% (73.0–78.5)	25.7% (22.9–28.8)	85.3% (79.4–90.1)	28.4% (21.9–35.7)	77.3% (74.8–79.8)	26.2% (23.6–29.0)
Specificity to diagnose moderate ICP	38.5% (36.0–40.9)	96.2% (95.1–97.1)	38.2% (35.3–41.0)	94.5% (93.0–95.8)	38.3% (36.5–40.2)	95.5% (94.6–96.3)
Positive predictive value to diagnose moderate ICP	43.4% (41.0–45.8)	81.0% (75.9–85.4)	18.2% (15.7–21.0)	45.0% (35.6–54.8)	34.8% (33.0–36.7)	71.0% (66.2–75.4)
Negative predictive value to diagnose moderate ICP	71.9% (68.7–74.9)	67.3% (65.2–69.4)	94.2% (91.6–96.1)	89.3% (87.4–91.0)	79.9% (77.6–82.1)	75.5% (74.0–77.0)
ROC AUC	0.57 (0.55–0.59)	0.61 (0.59–0.63)	0.62 (0.59–0.65)	0.62 (0.58–0.65)	0.58 (0.56–0.59)	0.61 (0.60–0.62)
Sensitivity to diagnose severe ICP	76.0% (71.1–80.4)	48.4% (42.9–54.1)	85.7% (73.8–93.6)	46.3% (32.6–60.4)	77.3% (72.9–81.4)	48.1% (43.0–53.3)
Specificity to diagnose severe ICP	34.4% (32.4–36.5)	93.7% (92.5–94.7)	35.8% (33.2–38.5)	93.1% (91.5–94.4)	34.9% (33.3–36.5)	93.4% (92.5–94.3)
Positive predictive value to diagnose severe ICP	15.5% (13.8–17.4)	55.9% (49.9–61.8)	5.6% (4.1–7.3)	22.5% (15.1–31.4)	12.1% (10.9–13.5)	46.4% (41.4–51.5)
Negative predictive value to diagnose severe ICP	90.0% (87.8–92.0)	91.7% (90.4–92.9)	98.3% (96.6–99.2)	97.5% (96.5–98.4)	93.0% (91.4–94.3)	93.9% (93.0–94.7)
ROC AUC	0.55 (0.53–0.58)	0.71 (0.68–0.74)	0.61 (0.56–0.66)	0.70 (0.63–0.76)	0.56 (0.54–0.58)	0.71 (0.68–0.73)

Moderate and severe ICP were determined by bile acid concentrations ≥40 and 100 µmol/L, respectively at the same time as measurement of ALT (alanine aminotransferase) and/or bilirubin. Patients receiving rifampicin and/or cholestyramine at the time of blood sampling were excluded from the analysis. Calculated results are presented with 95% confidence interval. *p < 0.0001 comparing rates between patients receiving and not receiving UDCA, calculated using Fisher's exact test. For moderate disease, calculations of sensitivity/specificity/positive predictive value/negative predictive value included all results ≥40 µmol/L (i.e. those also with severe disease).

### Identification of predictors of disease severity at diagnosis

Three hundred and eighty-eight individuals had BA concentration <40 µmol/L at diagnosis (mild disease), of whom 226 (58.3%) developed moderate-severe disease (BA ≥40 µmol/L). BA concentration at diagnosis was <100 µmol/L for 459 patients, of whom 107 (23.3%) developed severe disease. Of the individual predictors, an earlier gestation of ICP diagnosis and higher BA concentration at diagnosis best predicted subsequent moderate disease, and a previous pregnancy not affected by ICP reduced this likelihood (Supplemental Table 2). The best predictors for severe disease were gestation of ICP diagnosis, BA concentration at diagnosis and the presence of additional liver disease, gallstones or gallbladder sludge on ultrasound. With multiple regression, the diagnosis gestation and BA concentration at diagnosis improved the predictive model for severe disease (combined predictor named ‘pred100’: ROC AUC 0.68, 95%CI 0.62–0.73). Whilst adding previous ICP history slightly improved the model for moderate disease (*p* = 0.048), as this is only relevant for multiparous patients, we decided to limit the model to the same two predictors (named ‘pred40’: ROC AUC 0.64, 95% CI 0.58–0.69) (Supplemental Figure 2, Supplemental Table 3). External validation of these predictors gave ROC AUC 0.70 (95% CI 0.65 to 0.76) for pred40 (n = 391) and ROC AUC 0.69 (95% CI 0.63 to 0.76) for pred100 (n = 468) (Supplemental Figure 2). The probability that participants later developed moderate or severe ICP was lower for the PITCHES validation cohort than would have been expected using the pred40 or pred100 scores (probability <0.0001 for both, two-sided tests) (Supplemental Figure 2); this may reflect a later median randomisation gestation (34.4 weeks') to the discovery cohort (median 30.4 weeks for diagnostic sample).

## Conclusion

Whilst there is a gradual increase in BA, ALT and bilirubin concentrations with advancing gestation following ICP diagnosis, the individual pattern of disease varies markedly. For patients who received treatment with UDCA, those with higher starting BA were more likely have falling BA concentrations, whilst they were more likely to rise if starting at lower levels. Although potentially due to a reversion towards the mean with repeated measures, it is plausible that UDCA presence when hypercholanaemia is mild could increase total measured concentrations and UDCA,^6^ whilst its choleretic properties might enhance BA excretion where elimination is impaired in more severe disease.^12^

Neither ALT nor bilirubin concentrations are both sensitive and specific enough to use as surrogate markers of concurrent BA concentrations, however the high negative predictive value of normal ALT suggests that it can be used as a ‘rule out test’ for severe disease whilst BA results are awaited in patients not receiving UDCA. For patients whose BA are mild or mild-moderate at diagnosis, an earlier gestation at diagnosis and higher BA concentration at this point are the best predictors of subsequent moderate or severe disease, although, these predictors are of limited benefit.

This study benefits from a large cohort of recruited patients, with multiple serial timepoints of measurement and richness of data to allow for stratified assessment of disease course. As an observational study, it was not designed or powered to determine whether UDCA specifically alters BA trajectory; as participants who did not receive UDCA had milder disease than those receiving UDCA, the two groups are not easily comparable. However, that the trajectory of BA over time increases for those with mild disease following UDCA treatment, and as the benefits of UDCA to reduce adverse perinatal outcomes are most evident for the reduction in spontaneous preterm birth for those patients with moderate-severe disease, so limiting UDCA treatment to those whose BA increase above 40 µmol/L may be appropriate, particularly where symptomatic benefit does not occur.

Whilst our findings cannot definitively predict disease course of ICP, they support some evidence-based counselling of risks for more severe disease. As antenatal disease management is stratified by peak BA concentration, so repeated BA measurement is therefore still required. Given variable availability and speed of BA assays in different setting, this study highlights the need for improved access to these tests to enable their application to clinical decision-making in real time. Furthermore, alternative biomarkers for disease severity or ICP-related adverse pregnancy outcomes may be beneficial, to improve the predictive models that we have developed above.

## Supplemental Material

sj-docx-1-obm-10.1177_1753495X251394433 - Supplemental material for Determining and predicting biochemical disease trajectory in intrahepatic cholestasis of pregnancy: A longitudinal cohort study Supplemental material, sj-docx-1-obm-10.1177_1753495X251394433 for Determining and predicting biochemical disease trajectory in intrahepatic cholestasis of pregnancy: A longitudinal cohort study by Nicholas J Williamson, Jenna Sajous, Tim IM Korevaar, Paul T Seed, Jenny Chambers, Peter H Dixon, Lucy C Chappell, Catherine Williamson and Caroline Ovadia in Obstetric Medicine
