# Supplementary material for: Determining and predicting biochemical disease trajectory in intrahepatic cholestasis of pregnancy: A longitudinal cohort study
Source: Obstet Med. 2025 Dec 9:1753495X251394433. Online ahead of print. doi: 10.1177/1753495X251394433 (PMC12689345; doi:10.1177/1753495X251394433)
Supplement: sj-docx-1-obm-10.1177_1753495X251394433 - Supplemental material for Determining and predicting biochemical disease trajectory in intrahepatic cholestasis of pregnancy: A longitudinal cohort study [file sj-docx-1-obm-10.1177_1753495X251394433.docx]

**Supplementary Table 1. Maternal demographics and gestational disease characteristics for those contributing to the longitudinal studies of disease course in ICP**

|  | UDCA treatment N = 390 | | No UDCA treatment N = 64 | |
| --- | --- | --- | --- | --- |
| Age at diagnosis (years)  median (IQR) | 32.0  (29.0 to 35.0) | N = 390 | 32.0  (30.0 to 35.0) | N = 64 |
| Body mass index (kg/m^2^)  median (IQR) | 24.0  (20.9 to 28.0) | N = 139 | 24.3  (22.2 to 27.7) | N = 64 |
| Ethnicity % (number)  White  Asian  Black  Mixed  Other | 74.4 (290)  15.1 (59)  4.1 (16)  3.6 (14)  2.8 (11) | N = 390 | 62.5 (40)  21.9 (14)  6.3 (4)  6.3 (4)  3.1 (2) | N = 64 |
| Parity % (number)  0  1  2  3  ≥4 | 56.2 (219)  29.0 (113)  9.5 (37)  3.3 (13)  2.1 (8) | N = 390 | 54.7 (35)  34.4 (22)  7.8 (5)  1.6 (1)  1.6 (1) | N = 64 |
| Multifetal pregnancy % (number) | 11.8  (46) | N = 390 | 9.4  (6) | N = 64 |
| Pre-eclampsia % (number) | 7.6 (25) | N = 329 | 1.6 (1) | N = 64 |
| Gestational diabetes mellitus % (number) | 14.5  (39) | N = 269 | 7.8 (5) | N = 64 |
| Cholelithiasis % (number) | 23.1 (90) | N = 390 | 14.1 (9) | N = 64 |
| Previous ICP (multiparous patients only) % (number) | 84.8  (145) | N = 171 | 82.8  (24) | N = 29 |
| Gestation of ICP diagnosis (weeks^+days^) median (IQR) | 30^+0^  (24^+5^ to 32^+3^) | N = 390 | 32^+5^  (28^+6^ to 34^+3^) | N = 64 |
| Bile acid peak (µmol/L) median (IQR) | 75  (42 to 137) | N = 390 | 27  (17 to 46) | N = 64 |
| ALT peak (IU/L)  median (IQR) | 166  (69 to 329) | N = 390 | 82  (39 to 175) | N = 63 |
| Bilirubin peak (µmol/L) median (IQR) | 12  (8 to 18) | N = 390 | 9  (7 to 12) | N = 62 |
| Gestation at birth  (weeks^+days^) median (IQR) | 37^+0^  (35^+2^ to 37^+5^) | N= 390 | 38^+0^  (37^+1^ to 38^+5^) | N = 64 |
| Preterm birth  % (number) | 45.9  (179) | N = 390 | 23.4  (15) | N = 64 |
| Spontaneous preterm birth % (number) | 11.2  (43) | N = 383 | 9.7  (6) | N = 62 |
| Birthweight centile  median (IQR) | 61.1  (35.8 to 78.6) | N = 427 | 60.1  (36.3 to 81.8) | N = 66 |
| Caesarean birth  % (number) | 43.9  (170) | N = 387 | 34.9  (22) | N = 63 |
| Estimated blood loss (ml)  median (IQR) | 400  (300 to 600) | N = 328 | 400  (300 to 600) | N = 57 |
| Meconium-stained amniotic fluid % (number) | 21.6  (73) | N = 338 | 15.8  (9) | N = 57 |
| Apgar score at 5 mins  median (IQR) | 10  (9 to 10) | N = 373 | 10  (9 to 10) | N = 59 |
| Neonatal unit admission % (number) | 35.1  (147) | N = 419 | 9.4  (6) | N = 64 |
| Stillbirth % (number) | 1.8 (8) | N = 438 | 1.4 (1) | N = 70 |

N: number, IQR: interquartile range, ALT: alanine aminotransferase. Cholelithiasis refers to ultrasound evidence prior to, or during, the index pregnancy of gallstones or biliary sludge. Results from each baby have been included separately in the birth outcome data for multifetal pregnancies. Birthweight centile calculated using INTERGROWTH calculator

**Supplementary Table 2. Ability of baseline patient characteristics to predict subsequent moderate or severe intrahepatic cholestasis of pregnancy for those with initial mild disease**

|  | Prediction of later moderate ICP  (bile acid concentration ≥ 40 µmol/L) | | Prediction of later severe ICP  (bile acid concentration ≥ 100 µmol/L) | |
| --- | --- | --- | --- | --- |
| Predictive variable | Chi^2^ statistic | p value | Chi^2^ statistic | p value |
| Maternal age | 1.61 | 0.205 | 0.19 | 0.664 |
| Body mass index | 1.88 | 0.171 | 0.06 | 0.802 |
| Ethnicity | 2.52 | 0.471 | 5.46 | 0.141 |
| Multifetal pregnancy | 0.02 | 0.887 | 0.04 | 0.846 |
| Previous liver disorder | 0.33 | 0.564 | 5.67 | 0.017 |
| Gallbladder disease | 3.41 | 0.182 | 6.00 | 0.050 |
| Assisted conception | 0.37 | 0.544 | 0.82 | 0.365 |
| Gestation at ICP diagnosis | 13.18 | <0.001 | 22.70 | <0.001 |
| Bile acid concentration | 11.08 | <0.001 | 11.58 | <0.001 |
| ALT concentration | 3.61 | 0.057 | 1.90 | 0.168 |
| Bilirubin concentration | 2.22 | 0.136 | 1.60 | 0.206 |
| Previous pregnancy with/without ICP | 6.33 | 0.042 | 3.19 | 0.203 |

**Supplementary Table 3. Use of predictive models at diagnosis to give a probability of later development of moderate or severe intrahepatic cholestasis of pregnancy**

| Pred40 model to predict subsequent moderate ICP | | Pred100 model to predict subsequent severe ICP | |
| --- | --- | --- | --- |
| Expected proportion with moderate ICP | Observed proportion with moderate ICP (n/N) | Expected proportion with severe ICP | Observed proportion with severe ICP (n/N) |
| 0 % | - | 0 % | 0 % (0/9) |
| 10 % | - | 10 % | 16 % (38/235) |
| 20 % | - | 20 % | 25 % (32/127) |
| 30 % | 28 % (5/18) | 30 % | 38 % (19/50) |
| 40 % | 46 % (40/87) | 40 % | 42 % (11/26) |
| 50 % | 58 % (72/124) | 50 % | 40% (4/10) |
| 60 % | 61 % (54/89) | 60 % | 60 % (3/5) |
| 70 % | 78 % (47/61) | 70 % | 0 % (0/1) |
| 80 % | 75 % (9/12) | 80 % | 100% (1/1) |
| 90 % | 100 % (1/1) | 90 % | - |

Results present proportions as percentages, and absolute numbers with each probability risk (divided by units of 10) from the total number of patients with that predictive model score – n: patients with subsequent moderate or severe disease, N: total number of patients with predictive score (Pred40 or Pred100) in that probability range.


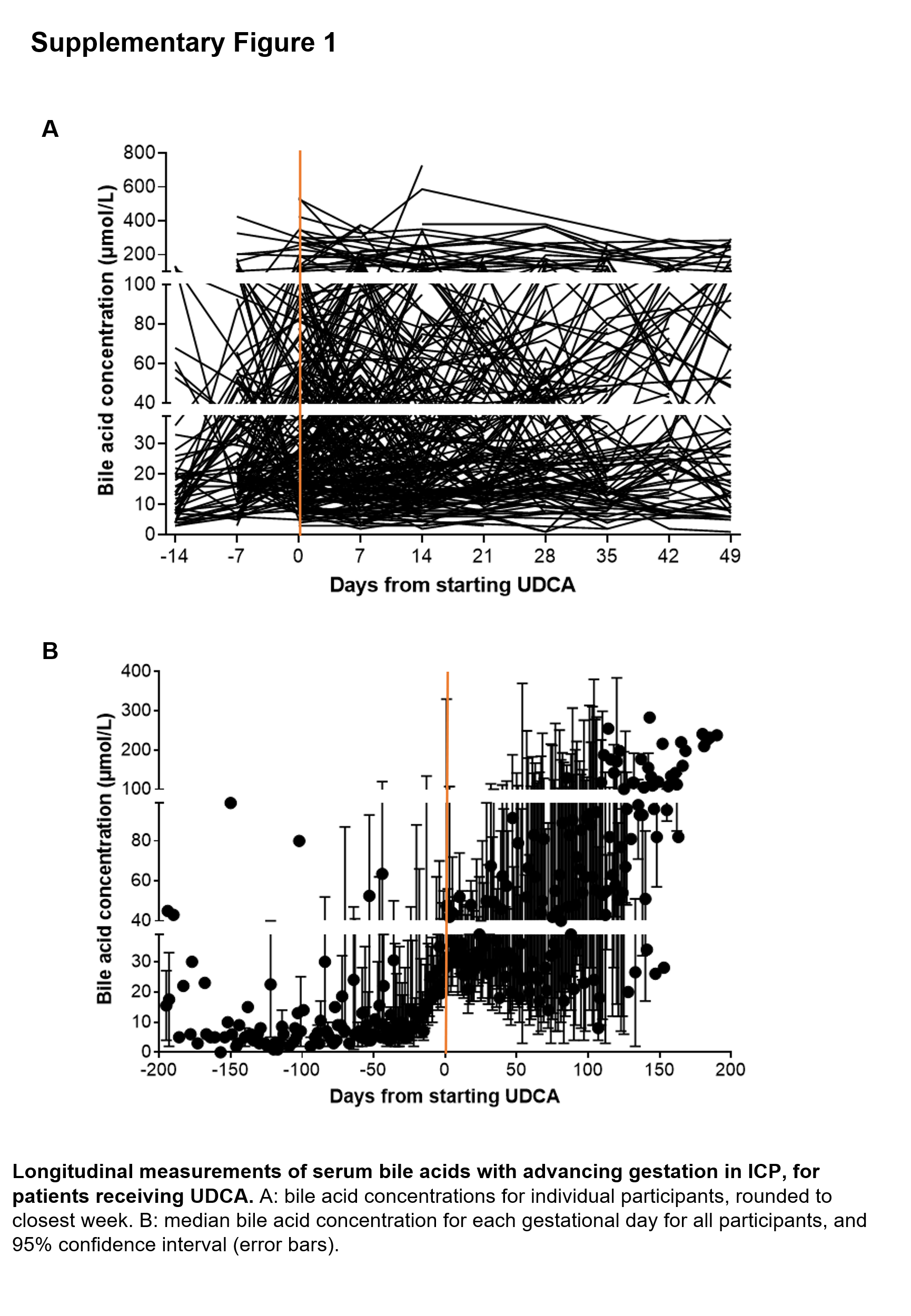


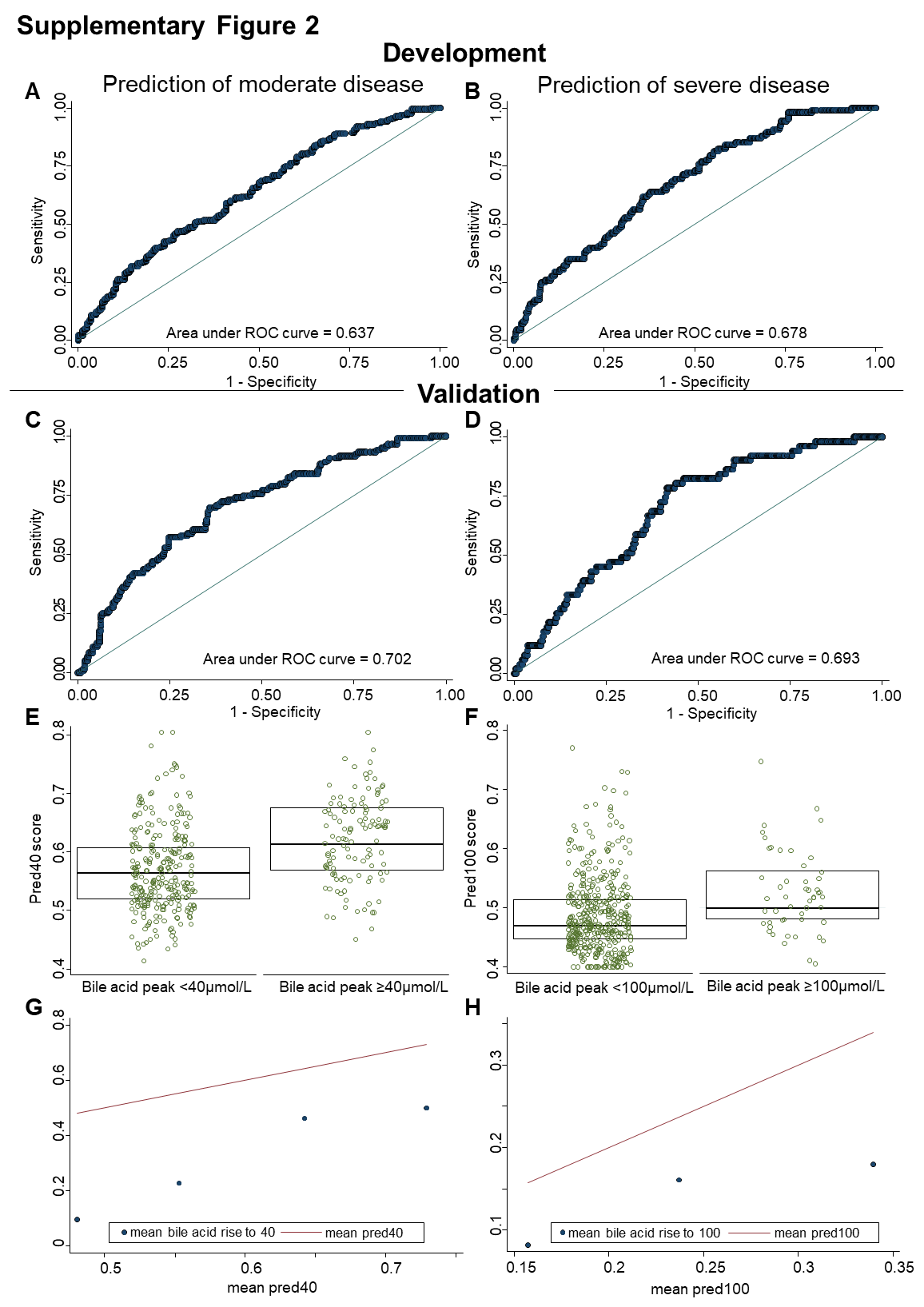


**Development of combined scored to predict subsequent moderate or severe disease. A**, **B** – Receiver operating curves of ability of prediction scores Pred40 (**A**) and Pred100 (**B**) to predict subsequent bile acids ≥40µmol/L and ≥100µmol/L, respectively, model developed from longitudinal observational cohort, incorporating gestational age at first bile acid measurement and initial bile acid concentration. **C**-**H**: Use of PITCHES trial patient cohort to validate prediction scores of subsequent moderate or severe disease**. C,D** – Receiver operating curves of ability of prediction scores Pred40 (**C**) and Pred100 (**D**) to predict subsequent bile acids ≥40µmol/L and ≥100µmol/L, respectively. **E**, **F**: prediction scores for patient groups by subsequent peak bile acid concentrations. **G**,**H**: observed (blue circles) versus expected (red line) proportion of patients with subsequent rise of bile acid concentrations above 40 (**G**) and 100µmol/L (**H**).
